# Expression of p53 in toluidine blue positive oral squamous cell carcinoma lesions and expression of Ki67 in vinegar positive oral squamous cell carcinoma lesions

**DOI:** 10.3389/froh.2023.1239961

**Published:** 2023-10-09

**Authors:** Kanokporn Bhalang, Kittipong Danuthai

**Affiliations:** ^1^ Department of Oral Medicine, Faculty of Dentistry, Chulalongkorn University, Bangkok, Thailand; ^2^ Department of Oral Pathology, Faculty of Dentistry, Chulalongkorn University, Bangkok, Thailand

**Keywords:** oral squamous cell carcinoma, oral cancer, toluidine blue, acetic acid, p53, Ki67

## Abstract

The aims of this study were to investigate the sensitivity and specificity of toluidine blue and/or vinegar in oral cancer screening and to examine the correlation between clinical screening using toluidine blue and vinegar and the expression of the tumor marker p53 and proliferation marker Ki67, respectively. The study consisted of 87 patients with suspected oral squamous cell carcinoma lesions. Toluidine blue and/or vinegar were applied to the lesions, followed by biopsies. The tissues were diagnosed histopathologically and underwent immunohistochemical process for p53 or Ki67. The results revealed that the sensitivity and specificity of oral cancer screening using toluidine blue were 93% and 46%, respectively; whereas the sensitivity and specificity using vinegar were 85% and 81%, respectively. A statistically significant correlation between the use of vinegar and the expression of Ki67 (*p* = 0.019) was observed. Although there was a difference in the expression of p53 between specimens that were positive and negative to toluidine blue, the correlation did not reach a significant level. Based on the results from this study, vinegar has a lower sensitivity than toluidine blue but a higher specificity for oral cancer screening. The results of the clinical screening using vinegar correlated with the expression of Ki67 at the cellular level.

## Introduction

Oral cancer is a global health problem (1). Oral squamous cell carcinoma (OSCC) is the most common cancer occurring in the oral cavity (2). In Thailand, the current five-year survival rate of 20%–30% is quite low (3). Early diagnosis is very important and can lead to improved survival rates. One diagnostic technique that is widely used internationally is the application of toluidine blue to suspected lesions (4, 5). The proposed mechanism of toluidine blue in early cancer detection is that toluidine blue is taken up by dysplastic cells, which have an increased density of nuclear material (6). Another potential early detection method is the use of 5% acetic acid, which has been utilized for cervical and oral cancer screening (7). These developments are more distinct in cancerous epithelium because it has high nuclear and protein concentration (9). A clinical screening technique would be more trustworthy if it correlates with cellular markers used for cancer detection. Several investigators suggest that protein p53, the product of a tumor suppressor gene, is one of the most interesting candidates for oral cancer detection (10, 11). p53 functions as a transcription factor controlling the cell cycle and the process of apoptosis (12), and disruptions in these result in cancer development. Mutation of *p53* changes the property of p53 protein resulting in its accumulation in the nucleus (13). A study has reported that toluidine blue positive cells have an allelic loss at chromosome 17p, which is the *p53* locus (14). It was revealed that p53 gene mutates late in carcinogenesis and could be associated with the invasive phenotype of oral squamous cell carcinoma (15). Thus correlating the expression of p53 expression and toluidine blue positive lesions might shed some light into the benefit of using the solution. Another marker to consider is Ki67, which functions to control cellular proliferation and is found only in proliferating cells (16). In normal epithelium Ki67 is found in the basal cell layer, but in malignant transforming tissue, Ki67 can be seen in every layer of the epithelium (17). The increase in number of genetic materials and protein in cancer tissues makes them positive to vinegar application can be confirmed by investigating the level of Ki67 protein. As the expression of Ki67 is significantly higher in tissues with poorer differentiated squamous cell carcinoma and more severe dysplasia (18), there is a prognostic value in using vinegar if it is correlated with the expression of Ki67.

Our previous study revealed that there was a difference in the expression of p53 in vinegar positive lesions (8). However, to the authors' knowledge, there has never been other studies that investigate the correlations between clinical screening of oral squamous cell carcinoma and immunohistochemically stained lesions. The aim of this study was to examine the application of toluidine blue and 5% acetic acid in the detection of oral premalignant lesions and to determine the association between clinical screening using toluidine blue and vinegar and the expression of p53 and Ki67, respectively.

## Materials and methods

### Study population

Eighty-seven patients with a clinical diagnosis of precancerous lesions or oral squamous cell carcinoma were recruited to participate in our study. They were from Rajvithi Hospital and Faculty of Dentistry, Chulalongkorn University Hospital, Bangkok, Thailand. The study was performed with informed consent and protocols approved by the Committees on Investigations Involving Human Subjects of the institutions.

### Clinical application of toluidine blue and vinegar

An oral medicine specialist recorded clinical findings, took pictures of the lesions, and picked the areas to be examined. Patients were then randomized into three interventions. Toluidine blue application was conducted on 33 patients. Five percent acetic acid (vinegar) application was conducted on a second group of 30 patients. Both toluidine blue and vinegar were applied to a third group of 24 patients.

For the application of toluidine blue, a cotton bud soaked in 1% acetic acid was used to clean the lesion prior to the application of toluidine blue with a different cotton tip for 30 s. Subsequently, a third cotton bud soaked with 1% acetic acid was used to remove any excess toluidine blue on the lesion. The patient then rinsed out their mouth. A positive lesion was the one which color changed to blue, while a negative lesion was with no change. The area was then photographed and an incisional biopsy was performed at the blue-stained area.

For acetic acid application, a gauze soaked with vinegar was applied to a clean and dry lesion for one minute. The researcher noted the changes and took picture of the lesion. A positive finding was designated when a lesion changed to opaque white. When a lesion did not change or changed to transparent white, a negative finding was indicated. An incisional biopsy was performed at the area that had turned opaque white.

For the 24 patients to which both substances were applied, application of vinegar was conducted first, followed by the application of toluidine blue. A positive finding was indicated for a lesion with color changed to both opaque white and blue, while a negative finding was a lesion not demonstrating these combined changes. An incisional biopsy was performed at the area that turned opaque white and had stained blue. If the areas that had stained blue and turned opaque white were not coincident, incisional biopsies were performed at both areas. If the lesion did not stain or change color, a biopsy was performed at the central area of the lesion.

### Immunohistochemical investigation

The specimen was routinely processed for histology, and sections were cut and then stained with hematoxylin and eosin for histopathological (final) diagnosis. For tissues obtained from patients receiving toluidine blue application, the next tissue section was used for the immunohistochemical study of tumor marker, p53 (19). For tissues obtained from patients receiving vinegar application, the next tissue section was used for the immunohistochemical study of proliferation marker, Ki67. A monoclonal antibody, anti-Ki67 (MIB 1, diluted 1:100; Dako, Denmark) was utilized as the primary antibody. The Envision plus kit (Dako, Denmark) was used for secondary antibody. The reaction was revealed by using 0.03% diaminobenzidine (DAB) solution. The Ki67 positive cells were counted under a light microscope at 400× magnification. The researcher picked three areas on each section for investigation. The cells were quantified by two researchers and then averaged. Positive controls were sections with known Ki67 overexpression. The negative control was performed with no primary antibody. The tissues from the 24 patients who had both substances applied on the lesions were not included in the immunohistochemical analysis.

### Statistical analyses

Sensitivity and specificity of toluidine blue and/or vinegar for oral cancer detection were calculated. The correlation between the results of the vinegar application and the final diagnoses was determined using Fisher's exact test. Differences in the percentage of p53/Ki67 positive cells between toluidine blue/vinegar positive and negative lesions were compared using the Mann–Whitney test. A *p*-value less than 0.05 was considered statistically significant.

## Results

As seen in Table 1, our study comprised 87 subjects (56% male/44% female) ranging in age from 25 to 86 years (average age 61.5 ± 12.38 years). Sample lesions were most commonly found on the lateral tongue (31.0%), buccal mucosa (20.7%), and floor of mouth (18.4%). Sixty-seven lesions received toluidine blue application, of which 58 were positively stained. Of these, 52 of were disease positive by histopathological diagnosis (Table 2). Of the 9 lesions negative to toluidine blue staining, 4 received a positive histopathological diagnosis. Thus, the sensitivity of toluidine blue for oral cancer detection was 92.86%, and the specificity was 45.45%. Vinegar was applied to 83 lesions, with 56 lesions showing positive results (Table 3). Upon histopathological diagnosis, 56 of these were seen to be disease positive. Of the 27 lesions negative to 5% acetic acid treatment, 9 of these were deemed disease positive. These results indicated that the sensitivity of vinegar for oral cancer detection was 85.25%, while the specificity was 81.82%. We applied both toluidine blue and 5% acetic acid to 27 lesions, finding 23 lesions positive to both substances (Table 4). Twenty-two of these were positive for disease by histopathological diagnosis. Negative staining results were observed for 5 lesions, 1 of which was found to be disease positive. The sensitivity and specificity when using both reagents for oral cancer screening were thus 95.65% and 80%, respectively. Using Fisher's exact test to evaluate the relationship between the results of oral cancer screening using toluidine blue or vinegar and the results of the final diagnoses showed significant correlations (*p* = 0.000 and *p* = 0.004, respectively).

**Table 1:** Characteristics of the patients.

Number of patients	87
Sex	Male 49 (56.3%)
	Female 38 (43.7%)
Male: Female	6.5: 5
Age range	25–86
Average age	61.5 ± 12.38
Location of lesions (each patient may have more than one lesion)	Lateral tongue 27 (31.0%)
	Buccal mucosa 18 (20.7%)
	Floor of mouth 16 (18.4%)
	Lower lip 9 (10.3%)
	Soft palate 7 (8.0%)
	Hard palate 5 (5.7%)
	Alveolar ridge 5 (5.7%)

**Table 2:** Results from toluidine blue (TB) application and the histopathological diagnoses (67 specimens from 54 patients).

	Histopathological diagnosis			
		Disease + ve^a^	Disease − ve^b^	Total
Toluidine blue application	+ ve result	52^c^	6	58
	− ve result	4	5^d^	9
	Total	56	11	67

^a^ Disease positive are dysplasia, carcinoma *in situ* and squamous cell carcinoma.

^b^ Disease negative are hyperplasia, inflammation and normal mucosa.

^c^ Sensitivity = 52/56 = 92.86%; Positive predictive value = 52/58 = 89.66%.

^d^ Specificity = 5/11 = 45.45%; Negative predictive value = 5/9 = 55.56.

**Table 3:** Results from 5% acetic acid application and the histopathological diagnoses (83 specimens from 57 patients).

	Histopathological diagnosis			
		Disease + ve^a^	Disease – ve^b^	Total
Acetic acid application	+ ve result	52^c^	4	56
	– ve result	9	18^d^	27
	Total	61	22	83

^a^ Disease positive are dysplasia, carcinoma *in situ* and squamous cell carcinoma.

^b^ Disease negative are hyperplasia, inflammation and normal mucosa.

^c^ Sensitivity = 52/61 = 85.25%; Positive predictive value = 52/56 = 92.86%.

^d^ Specificity = 18/22 = 81.82%; Negative predictive value = 18/27 = 66.67%.

**Table 4:** Results from both toluidine blue (TB) and 5% acetic acid application and the histopathological diagnoses (28 specimens from 24 patients).

	Histopathological diagnosis			
		Disease + ve^a^	Disease – ve^b^	Total
TB and acetic acid application	+ ve result	22^c^	1	23
	– ve result	1	4^d^	5
	Total	23	5	28

^a^ Disease positive are dysplasia, carcinoma *in situ* and squamous cell carcinoma.

^b^ Disease negative are hyperplasia, inflammation and normal mucosa.

^c^ Sensitivity = 22/23 = 95.65%; Positive predictive value = 22/23 = 95.65%.

^d^ Specificity = 4/5 = 80.00%; Negative predictive value = 4/5 = 80.00%.

**Figure 1:**
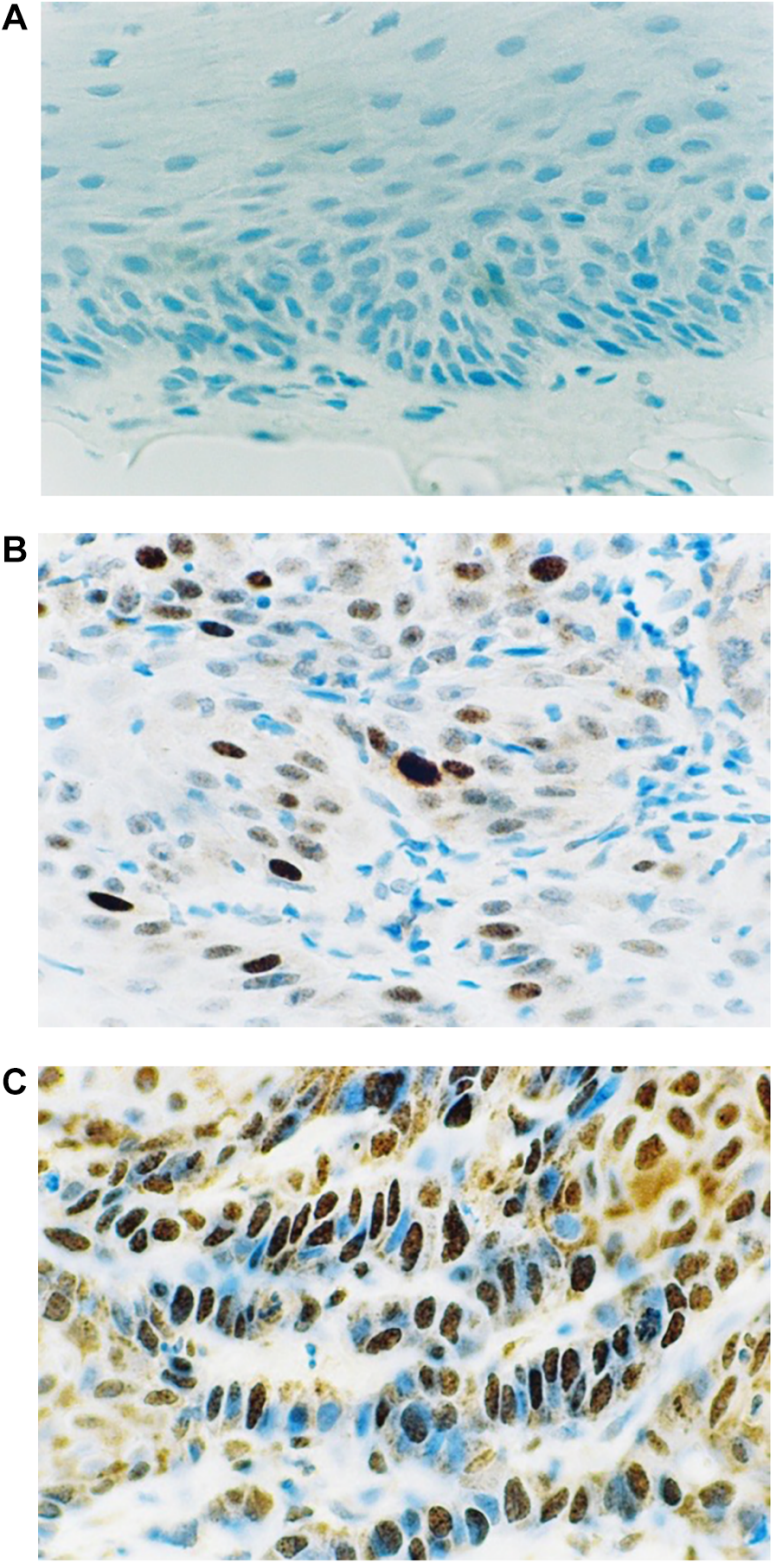
Immunohistochemical staining for p53. (**A**) Normal oral mucosa (**B**) epithelial dysplasia (**C**) oral squamous cell carcinoma.

**Figure 2:**
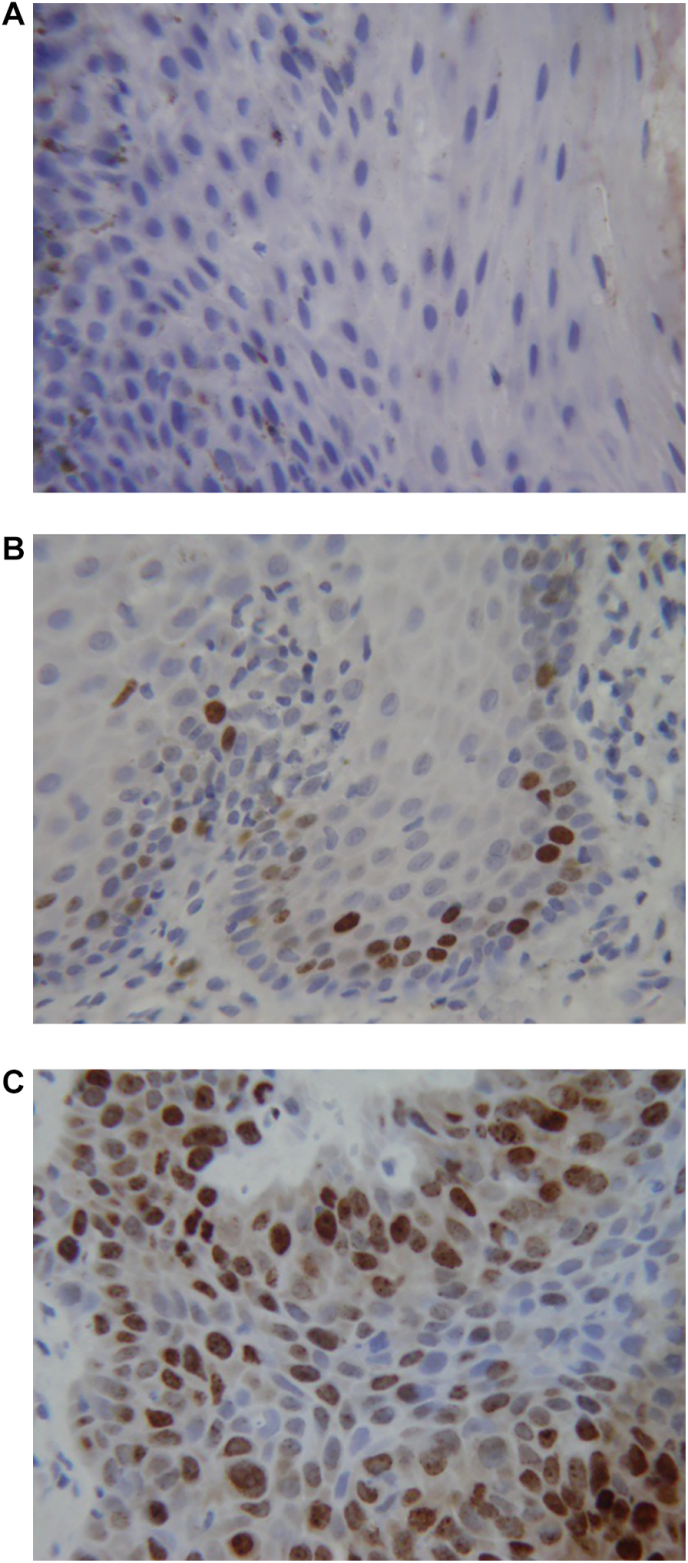
Immunohistochemical staining for Ki67. (**A**) Normal oral mucosa (**B**) epithelial dysplasia (**C**) oral squamous cell carcinoma.

**Figure 3:**
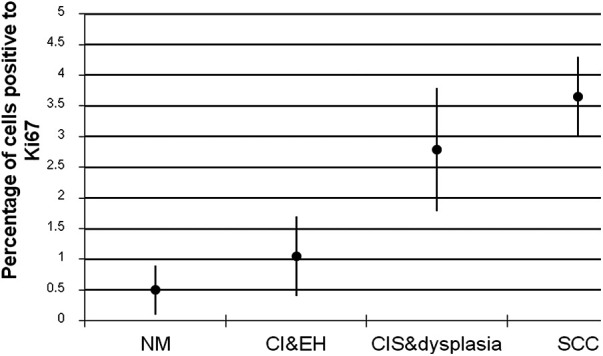
Percentage of cells positive to Ki67 antibody ± standard deviations in each group of specimens (NM, normal mucosa; CI, chronic inflammation; EH, epithelial hyperplasia; CIS, carcinoma *in situ*; SCC, squamous cell carcinoma).

Figure 1 shows the results of the immunohistochemical staining for p53. Normal mucosa demonstrated little to no observable staining (Figure 1A). Scattered cells with positive staining were noted in samples of dysplasia (Figure 1B). Oral squamous cell carcinoma samples displayed many positively stained cells arranged in cord-like structures, suggesting a clonogenic origin (Figure 1C). Although the percentage of cells with p53 staining in all toluidine blue positive specimens at 4.93 ± 1.32% was higher than that of the toluidine blue negative specimens at1.49 ± 0.97%, the difference between these two groups was not significant (*p* = 0.198) (Table 5). The results of the immunohistochemical staining for Ki67 are seen in Figure 2. Scant, if any, staining was noted in normal oral mucosa (Figure 2A), Dysplastic samples, however, showed robust staining in the suprabasal epithelial layer (Figure 2B). In contrast, in samples of oral squamous cell carcinoma widespread positive staining was seen throughout the epithelial cell layers (Figure 2C). We found increased numbers of specimens positive for Ki67 staining as the histopathological diagnosis rose in severity from normal mucosa (1/33%), to epithelial hyperplasia and chronic inflammation (4/44.4%), through epithelial dysplasia and carcinoma *in situ* (9/81.8%), and oral squamous cell carcinoma (24/88.9%) (Table 6). The percentage of stained cells based on severity was seen to follow the same trend (Figure 3). The positive cells for Ki67 in all vinegar positive specimens was 3.23 ± 0.58% and that of the vinegar negative specimens was 1.45 ± 0.45%, with this difference being significant (*p* = 0.018) (Table 7).

**Table 5:** Percentage of cells positive to p53 antibody by the results of toluidine blue application.

Percentage of cells positive to p53 ± standard errors	
Positive to toluidine blue application	Negative to toluidine blue application
4.93 ± 1.32	1.49 ± 0.97
*p* = 0.198^a^	

^a^ Analyzed by Mann–Whitney test.

**Table 6:** Number of specimen positive to Ki67 in each group of histopathological diagnosis.

Histopathological diagnosis (number of specimens)	Number of specimens that are positive to Ki67 (%)
Oral squamous cell carcinoma (27)	24 (88.9%)
Epithelial dysplasia and carcinoma *in situ* (11)	9 (81.8%)
Epithelial hyperplasia and Chronic inflammation (9)	4 (44.4%)
Normal Mucosa (3)	1 (33.3%)

**Table 7:** Percentage of cells positive to Ki67 antibody by the results of vinegar application.

Percentage of cells positive to Ki67 ± standard errors	
Positive to vinegar application	Negative to vinegar application
3.23 ± 0.58	1.45 ± 0.45
*p* = 0.019^a^	

^a^ Analyzed by Mann–Whitney test.

## Discussion

The patients in our study were 61.8 ± 11.8 years old with a male to female ratio of 6 to 5 is comparable with that of other studies (1, 20). Betel nut chewing is still common in the Thai elderly female population (8). This is consistent with higher number of female patients in our study. The lesion location distribution is also comparable to those found in other studies (1, 20), with the exception of a higher percentage of lesions on the buccal mucosa (23%) in our study.

When we compared the sensitivity and specificity of toluidine blue in the present study to the sensitivity and specificity of toluidine blue used for oral cancer screening from other studies (21–24), our results had a comparable sensitivity, but a much lower specificity. The reported low specificity here could stem from the low number of control lesions and the inclusion of all blue stained lesions. It has been reported that pale royal blue stained lesions are unrelated to any histological feature (25). The sensitivity and specificity of the vinegar used for oral cancer screening were higher when compared with the use for cervical cancer detection (7, 9, 26–28). When both substances were used on the same lesion, the sensitivity reached 96% and the specificity was as high as 80%.

The association between oral cancer screening using toluidine blue or vinegar and final diagnoses indicated that toluidine blue and vinegar reacted better with malignant or dysplastic tissues than with normal tissues, warranting the application of toluidine blue and 5% acetic acid in oral cancer screening. As vinegar has a comparable sensitivity to toluidine blue but a higher specificity in oral cancer screening, future study evaluating the use of vinegar for the detection of oral premalignant lesions in rural communities will be of value.

Our results revealed that the observable transformations due to vinegar use in oral cancer screening and the results of final diagnoses were associated with a significant difference in Ki67 positive cells between tissues that were positive to vinegar screening and those that were not. Although the number of p53 stained cells were higher in specimens that were positive to toluidine blue than those negative to toluidine blue, the difference did not reach a significant level. This could stem from the difficulty in separating lesions that are truly positive to toluidine blue (dark royal blue stain) and false positives (pale blue stain) as stated by a recent publication that pale blue lesions have no histological significance (25). The number of cells positive to Ki67 grouped by the final diagnoses showed an association between the advancement of the lesions and the level of Ki67 antibody. The average percentage of cells positive to Ki67 antibody in specimens of oral squamous cell carcinoma, carcinoma *in situ*, and epithelial dysplasia was significantly higher than less affected specimens. This revealed that the immunohistopathological diagnosis of oral premalignant and malignant lesions are correlated with the number of cells positive to Ki67 antibody. Our findings further warrant the application of both p53 and Ki67 antibody reaction for oral cancer treatment planning and prognosis determination.

## Data Availability

The raw data supporting the conclusions of this article will be made available by the authors, without undue reservation.
